# Inhibition of Melanoma Cells A375 by Carotenoid Extract and Nanoemulsion Prepared from Pomelo Leaves

**DOI:** 10.3390/plants10102129

**Published:** 2021-10-07

**Authors:** Man-Hai Liu, Yi-Fen Li, Bing-Huei Chen

**Affiliations:** 1 Department of Food Science, China University of Science and Technology, Taipei 11581, Taiwan; manhailiu@gmail.com; 2 Department of Food Science, Fu Jen Catholic University, New Taipei City 24205, Taiwan; kim06060515@gmail.com; 3 Department of Nutrition, China Medical University, Taichung 40401, Taiwan

**Keywords:** melanoma cell A375, carotenoid nanoemulsion, pomelo leaves, HPLC-MS

## Abstract

This study aims to determine carotenoids in pomelo leaves (*Citrus grandis* Osbeck), a rich source of nutrients and phytochemicals, by high-performance liquid chromatography-mass spectrometry and prepare carotenoid nanoemulsions for the study of its inhibitory mechanism on melanoma cells A375. Fourteen carotenoids were separated within 27 min by using a YMC-C30 column and a gradient mobile phase of methanol-acetonitrile-water (84:14:2, *v*/*v*/*v*) and methylene chloride with a flow rate of 1 mL/min and detection wavelength of 450 nm. All-trans-lutein plus its cis-isomers were present in the largest amount (3012.97 μg/g), followed by all-trans-neoxanthin (309.2 μg/g), all-trans-violaxanthin (208.5 μg/g), all-trans-β-carotene plus its cis-isomers (203.17 μg/g), all-trans-α-carotene plus its cis-isomers (152.5 μg/g), all-trans-zeaxanthin (54.67 μg/g), and all-trans-β-cryptoxanthin plus its cis-isomers (24.56 μg/g). A stable carotenoid nanoemulsion was prepared with a mean particle size of 13.3 nm, zeta-potential of −66.6 mV, a polydispersity index of 0.132 and an encapsulation efficiency of 99%. Both the carotenoid extract and nanoemulsion could upregulate p53, p21, cyclin B and cyclin A expressions in melanoma A375 cells and downregulate CDK1 and CDK2 in a concentration-dependent manner. Also, they could upregulate Bax and cytochrome-C and downregulate Bcl-2, leading to cell apoptosis through activation of caspase-9, caspase-8 and caspase-3. Compared to extract, carotenoid nanoemulsion was shown to be more effective in inhibiting the growth of melanoma cells A375. This finding further demonstrated that a carotenoid nanoemulsion prepared from pomelo leaves possessed a great potential to be developed into functional foods or even botanic drugs.

## 1. Introduction

Pomelo (*Citrus grandis* Osbeck), a kind of citrus plant belonging to *Rutaceae*, is mainly grown in Asian countries, such as India, Malaysia and Taiwan. Pomelo has been shown to be rich in dietary fibers, vitamins, minerals and phytochemicals, such as naringin and neohesperidin, both of which have been shown to effectively prevent chronic diseases, such as diabetes and atherosclerosis, as well as enhancement of immunity [1]. In addition, pomelo leaves, a waste product often discarded after pomelo harvest, was also shown to contain a high amount of bioactive compounds, such as carotenoids. Thus, it will be advantageous to the food industry if carotenoids can be extracted from pomelo leaves and developed into functional foods. 

Carotenoids, a group of lipid-soluble pigments widely distributed in nature especially green plants, can be classified into carotenes and xanthophylls, with the former being hydrocarbons and the latter oxygenated derivatives [2]. The biological activity of carotenoids has been well documented. For instance, carotenoids have been reported to possess strong scavenging activity of free radicals [3], enhance the immune system [4] and inhibit various types of cancers, such as prostate cancer [5], breast cancer [6], and colon cancer [7]. However, due to the presence of long-chain conjugated carbon-carbon double bonds, carotenoids can be prone to isomerization or degradation during heating, illumination, and exposure to oxygen or acid. Nevertheless, the antioxidant activity increases with the number of conjugated carbon-carbon double bonds in carotenoids [8]. Thus, it is imperative to employ an appropriate technique for encapsulation of unstable carotenoids to improve bioavailability and biological activity [2,8]. Among the various techniques, nanoemulsion or microemulsion is often chosen for carotenoid encapsulation because of the ease of preparation and the presence of ultra-small particles [9].

Accordingly, both nanoemulsion and microemulsion can be classified into three types: oil in water, water in oil and bi-continuous (both oil and water droplets are continuous phase). In addition, the selection of a suitable surfactant is a key step for successful preparation of a microemulsion or nanoemulsion [10]. Furthermore, surfactants can be classified into hydrophile or lipophile depending on the hydrophile-lipophile balance number (HLB), with HLB < 9 being lipophilic and HLB >11 being hydrophilic. Several non-ionic surfactants, such as Tween 80 and lecithin are frequently used due to low toxicity [11]. Also, co-surfactants and cosolvents can be used to reduce the level of surfactants for the preparation of nanoemulsion and microemulsion [10].

In addition to elevate bioavailability, both nanoemulsion and microemulsion can be used as a carrier to increase the water solubility of lipophilic drugs, improve the slow release of drugs in vivo, provide protection of easily hydrolyzable drugs in vivo, and strengthen penetration into barriers, such as blood-brain barrier or blood-eye barrier as well as cancer cells for cancer treatment [12]. For example, Huang, Wei, Inbaraj and Chen [7] prepared lycopene-nanogold nanoemulsion (21.3 nm) and demonstrated its inhibition efficiency against colon cancer cells HT-29 growth mainly by passive targeting through the enhanced permeability and retention (EPR) effect. In other words, the nanoemulsion should possibly be able to diffuse from the extracellular matrix into cytoplasm and nucleus to facilitate antitumor efficiency [7]. 

In view of the possible impact of microemulsion or nanoemulsion on cancer treatment, this study was undertaken to explore the inhibitory effects of carotenoid nanoemulsions (CNs) prepared from pomelo leaves on melanoma cells A375. In addition, the various carotenoids in pomelo leaves were analyzed by HPLC-MS, while carotenoid extracts (CEs) was compared with CNs for the inhibition efficiency of melanoma cells A375.

## 2. Results and Discussion

### 2.1. HPLC Analysis of Carotenoids in Pomelo Leaves

**Figure 1:**
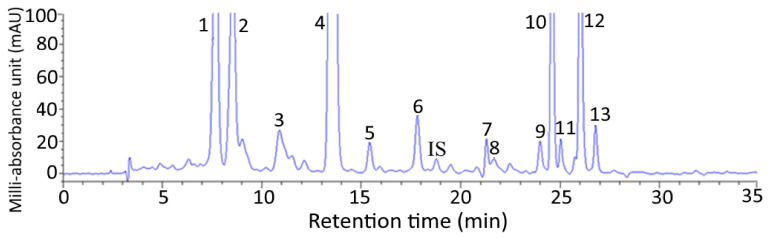
HPLC chromatogram of carotenoids isolated from pomelo leaves by using a C_30_ column and mobile phase of methanol-acetonitrile-water (84:14:2, *v*/*v*/*v*) (A), methylene chloride (B) with the following condition: 100% A in the beginning, decreased to 88% A in 2 min, 86% A in 8 min, 77% A in 14 min, 70% A in 19 min, 60% A in 21 min, 60% A in 24 min, 55% A in 34 min and finally 0% A in 38 min with flow rate at 1 mL/min and detection wavelength at 450 nm. Peak identification and quantification data are shown in Table 1.

Figure 1 shows a HPLC chromatogram of carotenoids extracted from pomelo leaves. Fourteen carotenoids including internal standard canthaxanthin were adequately separated within 27 min and identified with all-trans-lutein plus its cis isomers (9 or 9’-cis-lutein and 13 or 13’-cis-lutein) being present in the largest amount (3012.97 µg/g), followed by all-trans-neoxanthin (309.2 µg/g), all trans-violaxanthin (208.5 µg/g), all-trans-β-carotene plus its cis isomers (9- or 9’-cis-β-carotene and 13-or 13’-cis-β-carotene) (203.17 µg/g), all-trans-α-carotene plus its cis isomer (9 or 9’-cis-α-carotene) (152.5 µg/g), all-trans-zeaxanthin (54.67 µg/g) and all-trans-β-cryptoxanthin plus its cis isomers (9- or 9’-cis-β-cryptoxanthin (24.56 µg/g) (Table 1). The retention time (t_R_), retention factor (k), and separation factor (α) of various carotenoids in pomelo leaves are also shown in Table 1, with t_R_, k, and α ranging from 7.67–26.78 min, 1.28–7.00 and 1.02–1.47, respectively. The α value indicated that a satisfactory separation of various carotenoids in pomelo leaves was attained, while the k value implied that an appropriate solvent strength was maintained in the mobile phase. A total of 13 carotenoids were identified in pomelo leaves based on the comparison of identification data including mass to charge ratio (*m*/*z*), maximum absorption wavelength (λ_max_) and Q-ratio of each carotenoid with that of standard and those reported in the literature [13,14,15,16,17] (Table 1).

### 2.2. Nanoemulsion Characteristics

**Figure 2:**
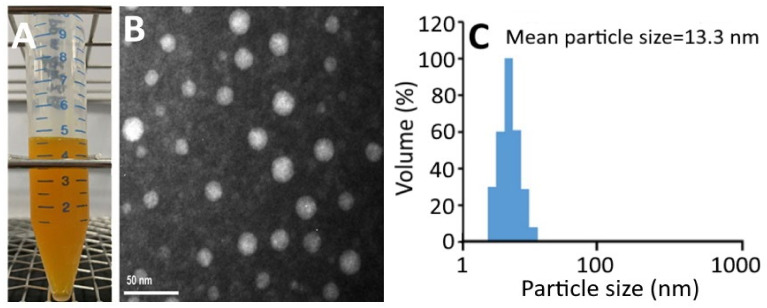
Carotenoid nanoemulsion from pomelo leaves (**A**) and its transmission electron microscope image showing round shaped particle with a mean particle size of 16 nm (**B**) as well as the particle size distribution obtained by DLS (**C**).

Figure 2A shows a transparent deep-yellow color of CNs, which should be due to the good dispersion of nanoparticles <100 nm without aggregation. The TEM image shows a round-shaped nanoparticle distribution with a mean particle size of 16 nm (Figure 2B), which is slightly larger than the average particle size (13.3 nm) obtained by DLS (Figure 2C). Figure S1A shows the changes in particle size, polydispersity index (PDI), zeta potential, and pH of CNs as affected by storage at 4 °C over a 3-month period. Only a slight change in zeta potential was observed for CNs during storage at 4 °C for 3 months, indicating a high stability of these CNs. According to a report by Yang, Hung and Chen [20], the zeta-potential of nanoemulsion should be >30 mV or <−30 mV to attain high stability. Similarly, only a minor increase in average particle size and PDI was shown in CNs during storage, revealing that a single distribution of nanoparticles was maintained. On the contrary, a slight decline in pH of CNs was shown during storage, which may be due to the hydrolysis of soybean oil into free fatty acid. A similar phenomenon was observed by Yang, Hung and Chen [20], who prepared coffee oil-algae oil-based nanoemulsions and a minor change in pH was shown over a 3-month storage period at 4 °C. However, during storage at 25 °C and 40 °C, a pronounced decline in pH could occur and resulted in lower nanoemulsion stability [20]. Thus, the control of storage temperature at 4 °C is a vital parameter to maintain high nanoemulsion stability and minimize lipid oxidation and hydrolysis at the same time. A similar outcome was shown by Qian, et al. [21] who encapsulated β-carotene into a nanoemulsion system composed of β-carotene, orange oil, Tween 20 and water. Figure S1B shows the changes in particle size and zeta-potential change of CNs as affected by heating at 40–100 °C for varied time lengths. Only a slight change in particle size of CNs was shown even after heating temperature and time reached 100 °C and 2 h, respectively, indicating a high thermal stability of these CNs. Conversely, the zeta-potential showed a pronounced temperature- and time-dependent decrease during heating, especially when the temperature reached 100 °C and 80 °C for 2 h, as evidenced by a drop of zeta-potential to −25.9 mV and −28.7 mV, respectively. This change would decrease the stability of CNs as the zeta-potential should be controlled at <−30 mV to maintain the high stability of the nanoemulsion [20].

### 2.3. Tolerance of Sample Solvent and Blank Nanoemulsion towards Cells

In this study, DMSO was used for cell culture, while both lecithin and Tween 80 were used for the preparation of CNs. To avoid interference of DMSO, lecithin and Tween 80 on the growth of melanoma cells A375 and fibroblast cells CCD-986SK, the tolerance of cells toward DMSO and blank nanoemulsion needs to be studied [22]. Based on a previous study by Liu, Li and Chen [22], the dose of DMSO and blank nanoemulsion was controlled at 1% to maintain the high viability of CCD-986SK and A375 cells for subsequent experiments.

### 2.4. MTT Assay

**Figure 3:**
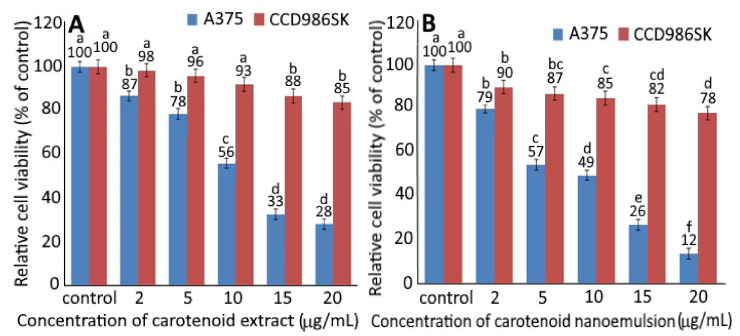
Relative cell viability of human melanoma cells A375 and fibroblast cells CCD986SK as affected by different concentrations of carotenoid extract (**A**) and carotenoid nanoemulsion (**B**) after 48 h incubation as measured by MTT assay. For control, cells were incubated in a medium containing 1% DMSO. Data shown are mean ± standard deviation (*n* = 3). Data with different small letters (a–f) in the same treatment but with different concentrations are significantly different (*p* < 0.05).

Figure 3 shows the relative cell viability of A375 cells and CCD986SK cells as affected by different concentrations of CEs (A) and CNs (B). A dose-dependent decline in cell viability was shown for both CCD-986SK and A375 cells upon treatment with CEs and CNs. With a concentration of CEs at 2, 5, 10, 15 and 20 µg/mL, the viability of CCD-986SK cells declined to 98, 96, 93, 88 and 85% as well as 90, 87, 85, 82 and 78% for CNs at the same dose. This result implied that CEs possessed relatively lesser toxicity towards CCD-986SK cells than CNs. However, both CEs and CNs showed slight toxicity on CCD-986SK cells. For A375 cells, the viability dropped to 87, 78, 56, 33 and 28% following treatment of CEs at 2, 5, 10, 15 and 20 µg/mL, respectively, as well as 79, 57, 49, 26 and 12% for CNs at the same dose. This phenomenon indicated that CNs exhibited higher toxicity on A375 cells than CEs, as evidenced by an IC_50_ of 4.2 and 11.8 µg/mL, respectively. It may be postulated that CNs should more easily penetrate into the cytoplasm and nucleus for A375 cell apoptosis [7]. Moreover, Dutta, et al. [23] demonstrated a synergistic inhibition effect on human esophageal epithelial cells and esophageal squamous cell carcinoma following treatment with α-carotene (5 μM) and β-carotene (5 μM) compared to α-carotene (10 µm) or β-carotene (10 µm) alone. In a similar study β-carotene (40 µm) was shown to be effective in inhibiting the growth of human esophageal squamous cell carcinoma EC9706 [24].

### 2.5. Cell-Cycle Analysis

From the MTT assay results, three concentrations (5, 10 and 20 µg/mL) of CEs and CNs were chosen to study the cell-cycle distribution and protein expression, as well as apoptosis-related proteins. An insignificant difference (*p* > 0.05) was observed in the sub-G1 phase between different CNs concentrations (5, 10 and 20 µg/mL) and control treatments (Table 2). Nevertheless, the ratio of the G2/M phase followed a concentration-dependent rise and attained 41.95% for CNs at 20 µg/mL, which was much higher compared to the control (26.27%). CEs also showed the same tendency, inferring that CEs or CNs at 10 and 20 µg/mL arrested the cell-cycle of melanoma cells A375 at G2/M phase. A similar outcome was shown for coffee oil-algae oil-based nanoemulsion, demonstrating the cell-cycle arrest for melanoma cells B16-F10 at G2/M phase with a dose at 0.04% [20]. Interestingly, Albino, et al. [25] reported that the cell-cycle of melanoma cells SK-Mel-29 and SK-Mel-110 was arrested at G0/G1 and S phases upon treatment with DHA standard at 0.5–2 µg/mL. Apparently, the cell-cycle arrest at a specific phase can vary depending on the cell type, as well as the dose and variety of nanoemulsions.

### 2.6. Expression of Cell-Cycle and Apoptosis-Related Protein

**Figure 4:**
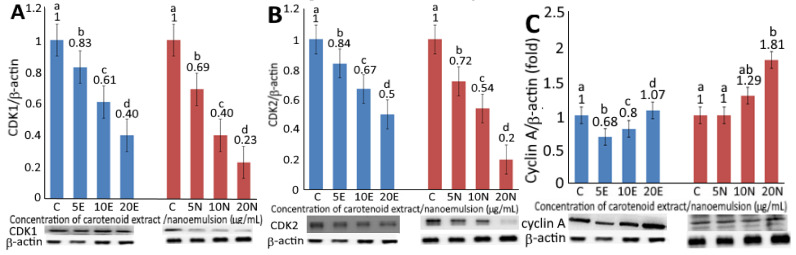

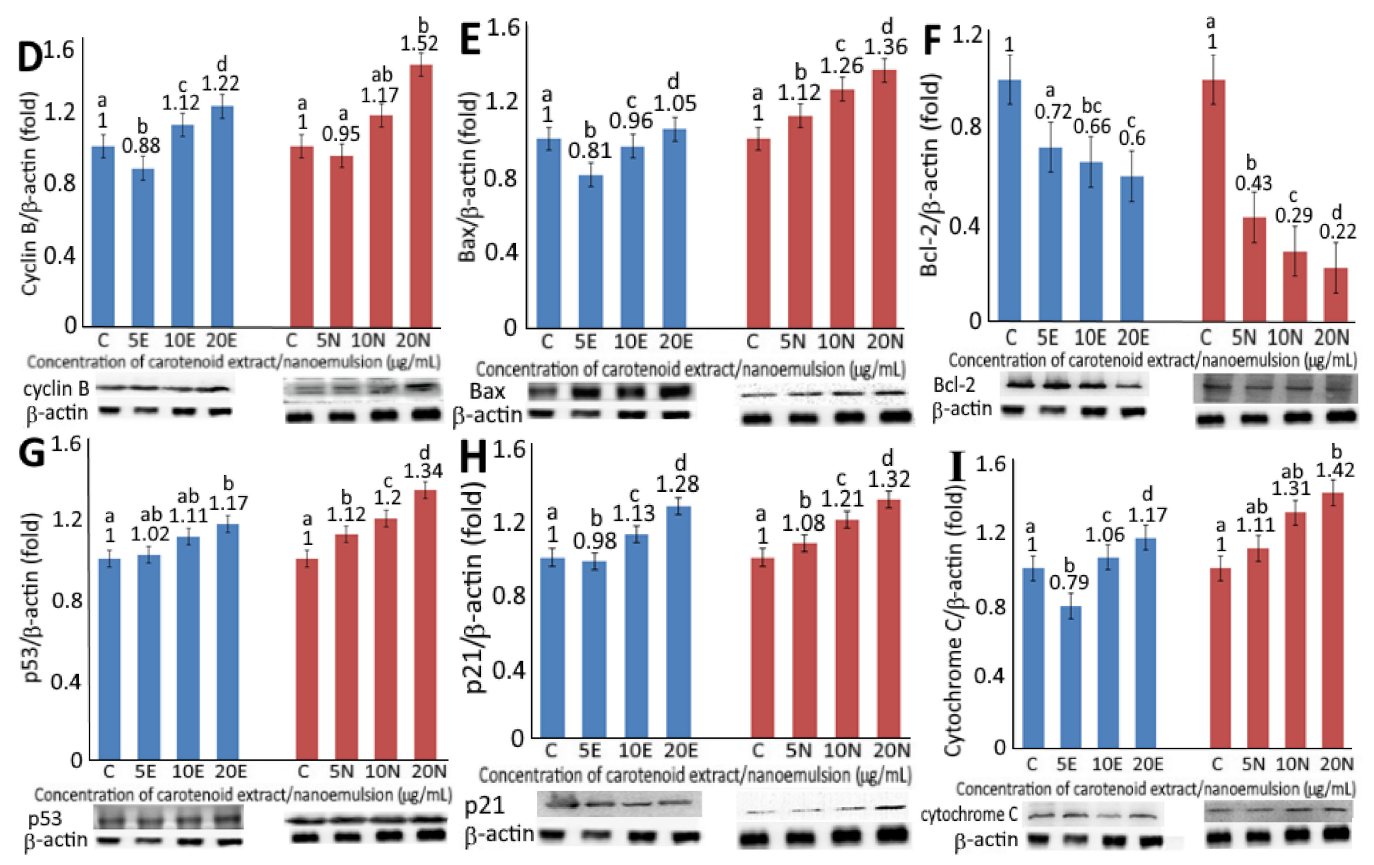
Levels of protein expressions for CDK1 (**A**), CDK2 (**B**), cyclin A (**C)**, cyclin B (**D**), Bax (**E**), BCL2 (**F**), p53 (**G**), p21 (**H**) and cytochrome C (**I**) as affected by carotenoid extract and nanoemulsion. For control, cells were incubated with medium only. Data are represented as mean ± standard deviation of triplicate determinations, with different small letters (a–d) denoting significantly different values at *p* < 0.05.

Upon treatment of CEs or CNs at 5, 10 and 20 µg/mL, a concentration-dependent decrease in expression of CDK1 (Figure 4A) and CDK2 (Figure 4B) was observed. By comparison at the same dose, CNs were more effective than CEs in decreasing expressions of CDK1 and CDK2. However, by comparison at the same treatment (5 and 10 µg/mL), the expression of CDK1 was slightly lower than CDK2. It has been well documented that nanoemulsion can improve the aqueous solubility of lipophilic bioactives, such as carotenoids to promote release and absorption in the gastrointestinal tract, thereby enhancing uptake by cells in vivo [7]. More recently, Mohd and Saima [26] pointed out that the application of polymeric nanoparticles for the encapsulation of herbal extracts could be a more efficient approach for the treatment of breast cancer through drug delivery. More importantly, it can be used as a targeted drug to remedy the problems associated with traditional drugs for the treatment of breast cancer, such as complications and side effects [26]. Thus, the CNs from pomelo leaves prepared in this study may possess a great potential to be developed into a botanical drug in the future.

The expression levels of cyclin A and cyclin B as affected by CEs and CNs are shown in Figure 4C,D, respectively. A concentration-dependent rise in cyclin A and cyclin B expressions was shown for both CEs and CNs. At the same dose, CNs were more effective than CEs in elevating both cyclin A and cyclin B expressions. Furthermore, following the treatment of CEs at the same dose, the expression level of cyclin B was higher than cyclin A, while a reversed trend was shown for CNs. As cyclin B possesses the tendency to form a complex with CDK1 for mitosis and G2/M phase regulation, the higher level of cyclin B implied that the cell-cycle of melanoma cells A375 might be arrested at the G2/M phase as shown above upon treatment of CEs or CNs. A similar finding was reported by Yang, Hung and Chen [20].

Bax, a member of the pro-apoptotic Bcl-2 family, can lower mitochondria membrane potential and release signals associated with apoptotic proteins, such as BCL-2 and cytochrome-C, resulting in the activation of caspase-9 and caspase-3 for subsequent cell apoptosis. Additionally, p53, a tumor suppressor protein, can increase Bax level via transcriptional regulation or conjugate with Bcl-2 to facilitate cell apoptosis [27]. A concentration-dependent rise in Bax expression was shown for both CEs and CNs (Figure 4E). At the same dose, CNs resulted in a higher Bax level than CEs, inferring that the former was more effective in promoting apoptosis of melanoma cells A375. Conversely, a concentration-dependent decrease in Bcl-2 levels was shown for both CEs and CNs (Figure 4F). At the same dose, CNs were more efficient in reducing Bcl-2 expression than CEs, demonstrating again a higher pro-apoptotic effect on melanoma cells A375 by CNs. Figure 4G shows p53 expression as affected by both CEs and CNs treatments, with the latter being more efficient in elevating p53 expression at the same dose. Also, a dose-dependent increase was shown for both CEs and CNs. An analog trend was observed for p21 expression (Figure 4H), with CNs being more effective in raising p21 level than CEs at the same dose, while a concentration-dependent rise in p21 expression was shown for both treatments. p21, a cyclin-dependent kinase inhibitor possessing the ability to inhibit cyclin/CDK complexes, is a major target of p53 activity responsible for linking DNA damage to cell-cycle arrest for tumor suppression [20]. Thus, it can be postulated that CNs should be more effective than CEs in inhibiting tumor formation. Like p53 and p21 expression, the cytochrome C expression in melanoma cells A375 as affected by both CEs and CNs showed the same trend, with the latter being more efficient than the former at the same dose, while a concentration-dependent increase was shown for both treatments (Figure 4I). Cytochrome C, a heme protein localized in the compartment between the inner and outer mitochondrial membranes, can be released into cytosol with a decrease in Bcl-2 activity for engagement of the apoptotic protease activating factor-1 (APAF1) and formation of the apoptosome for subsequent caspase-9 activation, also known as “initiator” for cell apoptosis [20].

**Figure 5:**
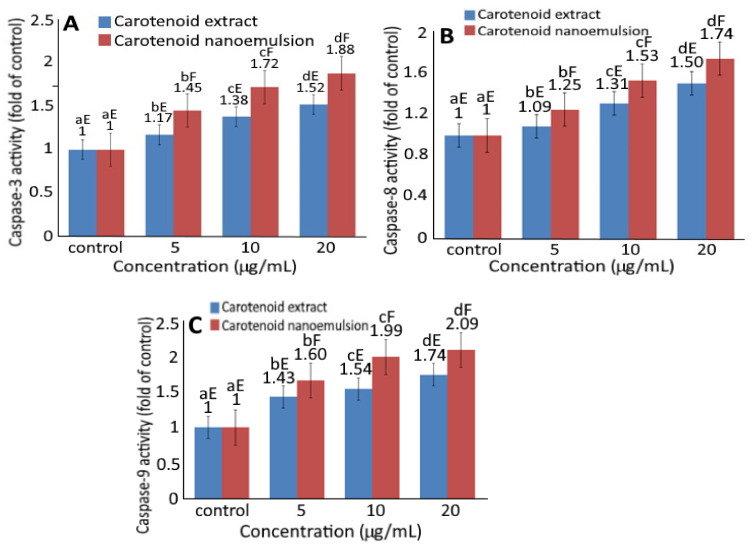
Effect of carotenoid extract and carotenoid nanoemulsion on the activities of caspase-3 (**A**), caspase-8 (**B**) and caspase-9 (**C**) in A375 cell line. Data are represented as mean ± standard deviation of triplicate determinations. For control, cells were incubated in a medium containing 1% DMSO. Data bearing different small letters (a–d) in the same treatment but with different concentrations as well as capital letters (E,F) in the same concentration but with different treatments are significantly different (*p* < 0.05).

Figure 5 shows the effect of CEs and CNs on the activities of caspase-3, caspase-8 and caspase-9. Upon treatment of melanoma cells A375 with CEs and CNs, a concentration-dependent rise in activities of caspase-3 (Figure 5A), caspase-8 (Figure 5B) and caspase-9 (Figure 5C) was shown. Also, at the same dose, CNs could result in higher activities of caspase-3, caspase-8 and caspase-9 than CEs.

Accordingly, cell apoptosis can be regulated through mitochondria, death receptor, or endoplasmic reticulum pathways with caspase-3 being activated through death receptor and mitochondria pathways, while caspase-8 through death receptor and caspase-9 through mitochondria [20]. As all the doses of both CEs and CNs were significantly higher (*p* < 0.05) than control, both death receptor and mitochondria pathways may be responsible for the apoptosis of melanoma cells A375.

In several previous studies Liu, et al. [28] reported that following treatment of retinal pigment epithelium cells with lutein (5–15 µm) for 24 h, the expressions of both CDK1 and CDC25C decreased, while a concentration-dependent increase in cyclin B expression occurred. Likewise, the expression of Bcl-2 mRNA decreased following treatment of cervical cancer cells HeLa with lutein at a dose of 1~10 µm [29]. Also, some other authors pointed out that carotenoids were effective in inhibiting the growth of acute myeloid leukemia cells via elevation of Bax, p53, cytochrome C and caspase-3 expressions [30]. All these findings suggested that the inhibition efficiency of carotenoids toward cancer cells could be dependent upon the type of cancer cells, as well as dose, variety and preparation method of CEs and CNs.

## 3. Materials and Methods

### 3.1. Materials

Twelve kilograms of pomelo leaves were obtained from a farm located at Hsin-Chu County, Taiwan, and were transported to our lab for freeze-drying, after which the pomelo leaves were divided into 10 bags with each containing 0.5 kg, sealed under vacuum and stored at −30 °C for use.

All the carotenoid standards were obtained from Sigma-Aldrich (St. Louis, MO, USA). HPLC-grade solvents were purchased from Lab-Scan Co. (Gliwice, Poland). Sigma-Aldrich also supplied the absorbents for column chromatography, such as magnesium oxide and diatomaceous earth. Anhydrous sodium sulfate, Tween 80 and lecithin were from Nacalai Tesque (Kyoto, Japan), Yu-Pa Co. (Taipei, Taiwan) and Chen-Fang Co. (Taipei, Taiwan), respectively.

### 3.2. Cell Culture and Reagents

All the materials dealing with cell culture including human fibroblast cells (CCD986SK) and human melanoma cells (A375), as well as reagents, media and caspase assay kits, were the same as described in a previous study [22].

### 3.3. Western Blotting Antibody

All the primary and secondary antibodies were the same as described in a previous study [22].

### 3.4. Instrumentation

All the instruments used in this study were the same as those reported by Liu, Li and Chen [22].

### 3.5. Extraction of Carotenoids from Pomelo Leaves

A method based on Inbaraj, Lu, Hung, Wu, Lin and Chen [13] was used to extract carotenoids from pomelo leaves with a slight modification. Dried pomelo leaf powder (1 g) was mixed with 30 mL of hexane-ethanol-acetone-toluene (10:6:7:7, *v*/*v*/*v*/*v*) in a flask, after which this mixture was shaken for 1 h and 2 mL of 40% potassium hydroxide in methanol was added, followed by flushing with nitrogen gas for subsequent saponification in the dark for 16 h. Then 15-mL of 10% sodium sulfate solution was added and shaken for 1 min, followed by collecting the supernatant, adding 15-mL hexane, shaking for 10 min and collecting the supernatant again. This step was repeated four times until the supernatant became colorless and finally, all the supernatants were pooled, dried, dissolved in 5 mL of methanol-methylene chloride (1:1, *v*/*v*), filtered through a 0.22-μm Nylon membrane filter and 20 μL injected into HPLC for analysis. All the extraction procedures were performed in the dark under nitrogen to prevent photooxidation, photodegradation and photoisomerization of carotenoids.

### 3.6. Preparation of Carotenoids from Pomelo Leaves by Open-Column Chromatography

A method based on Inbaraj, Lu, Hung, Wu, Lin and Chen [13] was modified to isolate carotenoids from pomelo leaves. To a 10-g sample of dried pomelo leave powder, 80 mL of hexane-ethanol-acetone-toluene (10:6:7:7, *v*/*v*/*v*/*v*) was added and shaken for 1 h, after which 80 mL hexane was added and shaken for 10 min to extract both carotenoids and chlorophylls. Next, 30 mL of anhydrous sodium sulfate (10%) was added for partition, and the upper layer containing carotenoids and chlorophylls was collected. Hexane (30 mL) was added to the bottom layer for further extraction and the supernatant was collected. This step was repeated four times until the supernatant became colorless. All the supernatants were combined, dried, dissolved in 10-mL hexane, and filtered through a 0.45 µm membrane filter to obtain crude extract. For open column chromatography, 0.5 mL of crude extract was poured into a glass column (325 × 24 mm ID) containing 10.5 g of magnesium oxide and diatomaceous earth at 1:2.5 (*w/w*). Then anhydrous sodium sulfate was added to form a layer of 1 cm, followed by adding 20 mL hexane for equilibrium and 25 mL of ethyl acetate/ethanol (98:2, *v*/*v*) for elution of carotenoids. The carotenoid eluate was used for the preparation of carotenoid nanoemulsions. Also, it was dried, dissolved in 1 mL of methanol/methylene chloride (1:1, *v*/*v*), filtered through a 0.22 µm membrane filter, and 20 µL injected into HPLC for qualitative and quantitative analyses of carotenoids.

### 3.7. HPLC Analysis of Carotenoids

A method based on Inbaraj, Lu, Hung, Wu, Lin and Chen [13] was modified to separate the various carotenoids in pomelo leaves by using a Waters YMC C30 column (250 × 4.60 mm ID, particle size 5 µm) and a mobile phase of methanol/acetonitrile/water (84:14:2, *v*/*v*/*v*) (A) and methylene chloride (B) with the following gradient elution: 100% A and 0% B, decreased to 88% A in 2 min, 86% A in 8 min, 77% A in 14 min, 70% A in 19 min, 60% A in 21 min, 55% A in 34 min and 0% A in 38 min. The flow rate was maintained at 1 mL/min and the detection wavelength at 450 nm. A total of 14 carotenoids including internal standard canthaxanthin were separated within 27 min and the separation efficiency was determined based on both retention factor (k) and separation factor (α).

Identification of various carotenoids in pomelo leaves was based on a comparison of retention time, absorption spectra and mass spectra of unknown peaks with those of reference standards as described in a previous study [13]. For further identification of cis isomers of carotenoids, the various carotenoid standards including lutein (100 µg/mL), β-carotene (100 µg/mL), β-cryptoxanthin (100 µg/mL), α-carotene (100 µg/mL) and zeaxanthin (50 µg/mL) were poured into a transparent 10-mL glass jar separately, followed by adding 100 µL of 0.01% iodine in methanol (*v*/*v*) and placing all the glass jars in an incubator (25 °C) for illumination for 1 h under 4 fluorescence light tubes (55 cm each) at a distance of 30 cm and light intensity at 2000–3000 Lux. After illumination, each carotenoid solution was filtered through a 0.22 µm membrane filter and 20 µL injected into HPLC for separation and identification of various all-trans and its cis isomers of carotenoids based on the identification criteria shown above [13]. Also, the purity of each peak was directly obtained from the G2180A Agilent spectral evaluation software system.

For quantification, six concentrations (0.6, 1, 5, 10, 20 and 50 µg/mL) of each carotenoid standard including all-trans-zeaxanthin, all-trans-β-cryptoxanthin, all-trans-lutein, all-trans-β-carotene and all-trans-α-carotene were prepared separately, and the internal standard canthaxanthin was added to each standard concentration for a final concentration at 10 µg/mL. After injection into HPLC, the calibration curve of each standard was prepared by plotting the concentration ratio of each carotenoid to internal standard against area ratio and their corresponding linear regression equation, as well as the coefficient of determination (r^2^) were obtained. The level of various carotenoids in pomelo leaves were quantified using a formula as described in a previous study [13].

### 3.8. Preparation of Carotenoid Nanoemulsion

A dried sample of CEs (2.5 mL) was mixed sequentially with 0.1 g soybean oil, 0.018 g vitamin E, 0.8 g Tween 80, 0.1 g lecithin and 8.982 g deionized water in the percentage ratio of 1:0.18:8:1:89.92 with intermittent homogeneous stirring after each addition. Then, the mixture was ultrasonicated for 1 h (4 °C) to obtain 10-mL transparent CNs containing carotenoid at 4.0 mg/mL. 

### 3.9. Determination of Carotenoid Nanoemulsion Characteristics

A 30-μL sample of CNs was diluted 100-fold with monopotassium phosphate solution (25 mM, pH 5.3–5.5) and filtered using a 0.45 µm membrane filter in a polystyrene tube for determination of particle size distribution and polydispersity index by a dynamic light scattering instrument (DLS). Similarly, a 10 μL sample of CNs was diluted 120-fold with deionized water for the determination of surface charge by a zeta potential analyzer. For the determination of particle size and shape, a transmission electron microscopic image (TEM) was recorded by dropping 20 μL of diluted sample onto a copper grid, followed by removing the excess sample with a glass filter paper, negative staining with 20 µL of 2% phosphotungstic acid (PTA) for 60 s, removing the excess PTA, drying completely in a desiccator, and observing the image under 120 kVa by enlarging 3 × 10^5^ times. Encapsulation efficiency (EE, %) of carotenoids in nanoemulsion were determined by collecting 100 μL of the sample, diluting 100-fold with monopotassium phosphate buffer (25 mM, pH 5.3–5.5) and pouring into a centrifuge tube equipped with a 3 kDa dialysis membrane for centrifugation at 12,000 rpm for 20 min (25 °C). Then, the EE was determined by analyzing the lower layer for free carotenoid by HPLC and substituting in the Formula shown below (1):

(1) $$\mathrm{EE}\;\left(\%\right)=\left(\frac{\mathrm{total}\;\mathrm{amount}\;\mathrm{of}\;\mathrm{carotenoid}-\mathrm{amount}\;\mathrm{of}\;\mathrm{free}\;\mathrm{carotenoid}}{\mathrm{total}\;\mathrm{amount}\;\mathrm{of}\;\mathrm{carotenoid}}\times 100\right)$$

### 3.10. Stability of Carotenoid Nanoemulsion

Storage stability of CNs was determined by collecting the sample stored at 4 °C every 15 days for measurement of particle size distribution and zeta potential over a period of 90 days. In addition, the appearance was observed to notice if there is any aggregation or cloudiness occurred during storage. Also, for stability during heating, a 200 µL sample of CNs was each placed in tubes, heated for 0, 5, 1, 1.5 and 2 h separately in a temperature-controlled water bath at 40, 50, 60, 70, 80, 90 and 100 °C and analyzed for both particle size distribution and zeta potential.

### 3.11. Cell Culture

Human melanoma cells A375 and fibroblast cells CCD-986SK were cultured in Eagle’s minimum essential medium (MEM) and Dulbecco’s modified Eagle’s medium (DMEM), respectively. Cells were thawed following collection from liquid nitrogen, followed by placing cell sap containing 7% DMSO on a cell culture plate, adding 10 mL of the MEM containing 10% FBS for incubation at 37 °C and 5% CO_2_ for subculture. After removal of medium, the cells were washed with PBS (2–3 times) and 1 mL of 0.25% trypsin-EDTA added for reaction (1–2 min) in an incubator, followed by adding 1 mL medium to terminate the reaction, centrifuging at 1000 rpm for 5 min (25 °C), removing the supernatant, adding 1 mL medium and collecting cell sap for subsequent culture experiments.

### 3.12. MTT Assay

A 96-well plate seeded with A375 cells (5 × 10^5^/well) were incubated for 24 h for cell detachment, followed by removing the medium, washing with PBS once and treating with different concentrations of CEs or CNs (2, 5, 10, 15 and 20 µg/mL) for performing triplicate experiments. After incubation for 72 h and eventual removal of the medium, as well as washing with PBS, 200 µL of MTT solution (0.5 mg/mL) was added for reaction in the dark for 1 h and 200 μL of DMSO added to dissolve purple crystals for measurement of absorbance at 570 nm in an ELISA reader. Then, the cell viability was calculated based on the following Formula (2):

(2) $$\mathrm{Cell}\;\mathrm{viability}\;\left(\%\right)=\left(\frac{\mathrm{treatment}-\mathrm{blank}}{\mathrm{control}-\mathrm{blank}}\times 100\right)$$

where, treatment = addition of CEs or CNs at different concentrations, blank = culture medium without cells, control = without sample treatment.

### 3.13. Cell-Cycle Analysis

A375 cells (5 × 10^6^/well) were seeded in a 6-well plate and cultured for 24 h. After cell adhesion, the medium was removed, different concentrations (5, 10 and 20 µg/mL) of CEs and CNs were added and incubated for 48 h. Then the cell-cycle distribution including the proportions of sub-G1, G0/G1, S and G2/M phases was analyzed by a flow cytometer following the same procedure as described in a previous study [22].

### 3.14. Western Blotting

Initially, a 10-cm plate seeded with 5×10^5^ cells was cultured for 24 h and subsequently, the medium was removed, washed with PBS and the medium replaced with 5, 10 and 20 µg/mL of CEs and CNs separately. Following the same procedure, as described in a previous study [22], the expressions of CDK1, CDK2, cyclin A, cyclin B, cytochrome-C, Bax, Bcl-2, p21 and p53 were determined.

### 3.15. Activities of Caspase-3, -8 and -9

A fluorometric assay-based kit was employed for the determination of caspase-3, caspase-8 and caspase-9 activities following the same procedures as described in a previous study [22].

### 3.16. Statistical Analysis

The statistical analysis system was used for performing statistical analysis of experimental data [31] and the data variance was analyzed by ANOVA and Duncan’s multiple range test for comparison of mean data for significance (*p* < 0.05).

## 4. Conclusions

Collectively, in our study, both carotenoid extract and nanoemulsion were effective towards inhibition of melanoma cells A375 via elevating cyclin A and cyclin B expressions and decreasing CDK1 and CDK2 expressions for cell-cycle arrest at the G2/M phase. Also, both p53 and p21 expressions were raised, resulting in an increase in Bax expression and a decline in Bcl-2 for cytochrome C release and complex formation with caspase 9, resulting in caspase-3 activation and apoptosis execution. Meanwhile, the activity of caspase-8 was enhanced for apoptosis initiation. Furthermore, a detailed in vivo study is necessary to elucidate the inhibition mechanism of carotenoid extract and nanoemulsion on tumors.

## Figures and Tables

**Table 1:** Retention time (t_R_), retention factor (k), separation factor (α), content (µg/g), *m/z*, λ_max_ and Q-ratio of various carotenoids in pomelo leaves.

Peak No.	Compound	t_R_ (min)	Retention Factor (k) ^a^	Separation Factor (α) ^b^	Content (μg/g) ^d^	*m/z* Found	*m/z* Reported	λ_max_ (nm, Online) ^j^	λ_max_ (nm, Reported) ^j^	Q-Ratio Found	Q-Ratio Reported
1	All-*trans*-neoxanthin	7.67	1.28	1.20 (1,2) ^c^	309.2	601.5 [M+H], 583 [M+H-18]	601.5 [M+H], 583 [M+H-18] ^f^	328, 418, 440, 470	330, 416, 442, 470 ^k^	0.08	-
2	All-*trans*-violaxanhin	8.53	1.53	1.47 (2,3)	208.5	601.5 [M+H], 583 [M+H-18]	601.5 [M+H], 583 [M+H-18] ^f^	328, 412, 436, 464	329, 416, 439, 469 ^g^	0.07	-
3	13-or 13’-*cis*- lutein	10.88	2.25	1.17 (3,4)	81.04	551.5 [M+H-18]	551.5 [M+H-18] ^g^	334, 426, 448, 470	334, 418, 441, 470 ^g^	0.35	0.38 ^l^
4	All-*trans*-lutein	12.13	2.62	1.16 (4,5)	2922.3	551.4 [M+H-18]	551.4 [M+H-18] ^g^	332, 440, 466	332, 445, 473 ^g^	0.07	0.07 ^l^
5	9-or 9’*cis*-lutein	13.54	3.04	1.18 (5,6)	9.63	551.4 [M+H], 533.4 [M+H-18-18]	551.4 [M+H], 533.4 [M+H-18-18] ^g^	332, 446, 474	332, 440, 468 ^g^	0.06	0.08 ^l^
6	All-*trans*-zeaxanthin	17.81	3.60	1.20 (6,IS)	54.67	569.4 [M+H], 551.4 [M+H-18]	569.4 [M+H],551.4 [M+H-18] ^g^	340, 452, 478	339, 452, 478 ^k^	0.07	0.06 ^l^
IS ^e^	All-*trans*-canthaxanthin	19.21	4.32	1.24 (IS,7)	-	-		478	-	-	-
7	All-*trans*-β-cryptoxanthin	21.29	5.36	1.02 (7,8)	18.63	553.4 [M+H], 535.4 [M+H-18]	553.4 [M+H], 535.4 [M+H-18] ^h^	456, 482	428, 450, 477 ^k^	0.14	0.16 ^m^
8	9-or 9’-*cis*-β-cryptoxanthin	21.66	5.47	1.13 (8,9)	5.93	553.4 [M+H]	553.4 [M+H] ^h^	336, 442, 468	332, 410, 440, 468 ^k^	0.12	0.11 ^m^
9	13-or 13’-*cis*-β -carotene	23.99	6.16	1.03 (9,10)	12.64	537.4 [M+H] ^g^	537.4 [M+H] ^g^	342, 448	332, 416, 440, 470 ^k^	0.42	0.43 ^m^
10	All-*trans*-α-carotene	24.56	6.33	1.02 (10,11)	142.6	537.3 [M+H]	537.3 [M+H] ^i^	450, 478	338, 418, 440, 472 ^k^	0.08	-
11	9-or 9’-*cis*-α-carotene	25.03	6.47	1.05 (11,12)	9.9	537.3 [M+H]	-	444, 472	440, 468 ^k^	0.10	-
12	All-*trans*-β-carotene	26.01	6.77	1.03 (12,13)	168.3	537.4 [M+H]	537.4 [M+H] ^g^	456, 482	442, 452, 476 ^k^	0.08	0.09 ^m^
13	9-or 9’-*cis*-β-carotene	26.78	7.00	1.03 (13,14)	22.23	537.4 [M+H]	537.4 [M+H] ^g^	452, 476	446, 448, 472 ^k^	0.13	0.13 ^m^

^a^ k = (t_R_ − t_0_)/t_0_, where t_R_ is the retention time of peaks while t_0_ is the retention time of solvent peak, ^b^ α = (t_R2_ − t_0_)/ (t_R1_ − t_0_) = k_2_/k_1_, where k_2_ is the retention factor of peak 2 and k_1_ is the retention factor of peak 1. ^c^ Numbers in parentheses represent peak numbers, ^d^ Average of duplicate analyses, ^e^ IS = internal standard, ^f^ Based on a report by Delgado-Pelayo, Gallardo-Guerrero and Hornero-Méndez [15], ^g^ Based on a report by Schex, et al. [18], ^h^ Based on a report by de Faria, Hasegawa, Chagas, Pio, Purgatto and Mercadante [14], ^i^ Based on a report by Petry and Mercadante [19], ^j^ λ_max_ = maximum absorption wavelength, ^k^ Based on report by Song, Li, He, Chen and Liu [17], ^l^ Based on a report by Liu, Chen, Kao and Shiau [16], and ^m^ Based on a report by Inbaraj, Lu, Hung, Wu, Lin and Chen [13].

**Table 2:** Cell-cycle distribution of melanoma cells A375 as affected by carotenoid extracts and nanoemulsions ^1,2^.

Concentration (µg/mL)	Sub-G1 (%)	G0/G1 (%)	S (%)	G2/M (%)
control	3.31 ± 0.06 ^B^	52.83 ± 1.41 ^A^	17.65 ± 3.27 ^A^	26.27 ± 1.54 ^A^
carotenoid extract				
5	4.03 ± 0.05 ^B^	49.36 ± 0.25 ^B^	16.23 ± 0.56 ^B^	28.15 ± 1.22 ^B^
10	4.25 ± 0.08 ^B^	47.78 ± 1.12 ^C^	15.86 ± 1.02 ^B^	31.36 ± 2.04 ^C^
20	3.91 ± 0.05 ^B^	46.48 ± 0.57 ^C^	15.23 ± 0.27 ^B^	35.27 ± 1.35 ^D^
carotenoid nanoemulsion				
5	4.36 ± 0.07 ^B^	45.27 ± 0.56 ^C^	15.57 ± 0.62 ^B^	32.86 ± 1.42 ^C^
10	4.18 ± 0.06 ^B^	44.53 ± 0.75 ^C^	16.14 ± 0.38 ^B^	36.72 ± 1.78 ^D^
20	4.98 ± 0.08 ^B^	42.35 ± 1.04 ^D^	14.92 ± 0.75 ^C^	41.95 ± 2.06 ^E^

^1^ Data shown are mean ± standard deviation. ^2^ Data with different capital letters (^A–E^) in the same column are significantly different at *p* < 0.05.

## Supplementary Materials

The following are available online at https://www.mdpi.com/article/10.3390/plants10102129/s1, Figure S1: Particle size, polydispersity index, pH and zeta potential of carotenoid nanoemulsion as affected by storage at 4 °C for 90 days (A) as well as both particle size and zeta potential of carotenoid nanoemulsion as affected by heating at different times and temperatures (B).
