# Supplementary material for: Inhibition of Melanoma Cells A375 by Carotenoid Extract and Nanoemulsion Prepared from Pomelo Leaves
Source: Plants (Basel). 2021 Oct 7;10(10):2129. doi: 10.3390/plants10102129 (PMC8539030; doi:10.3390/plants10102129)
Supplement: Supplementary file 1 [file plants-10-02129-s001.zip › plants-1393265-supplementary-corrected.pdf]

Supporting information for

Inhibition of Melanoma Cells A375 by Carotenoid Extract and Nanoemulsion Prepared from Pomelo Leaves

Man-Hai Liu[1], Yi-Fen Li[2] and Bing-Huei Chen[2,3,*]

[1]Department of Food Science, China University of Science and Technology, Taipei – 11581, Taiwan.
[2]Department of Food Science, Fu Jen Catholic University, New Taipei City – 24205, Taiwan.
[3]Department of Nutrition, China Medical University, Taichung – 404, Taiwan.
* Correspondence: 002622@mail.fju.edu.tw; Tel.: +886 2 2905 3626; Fax +886 2 2209 3271.

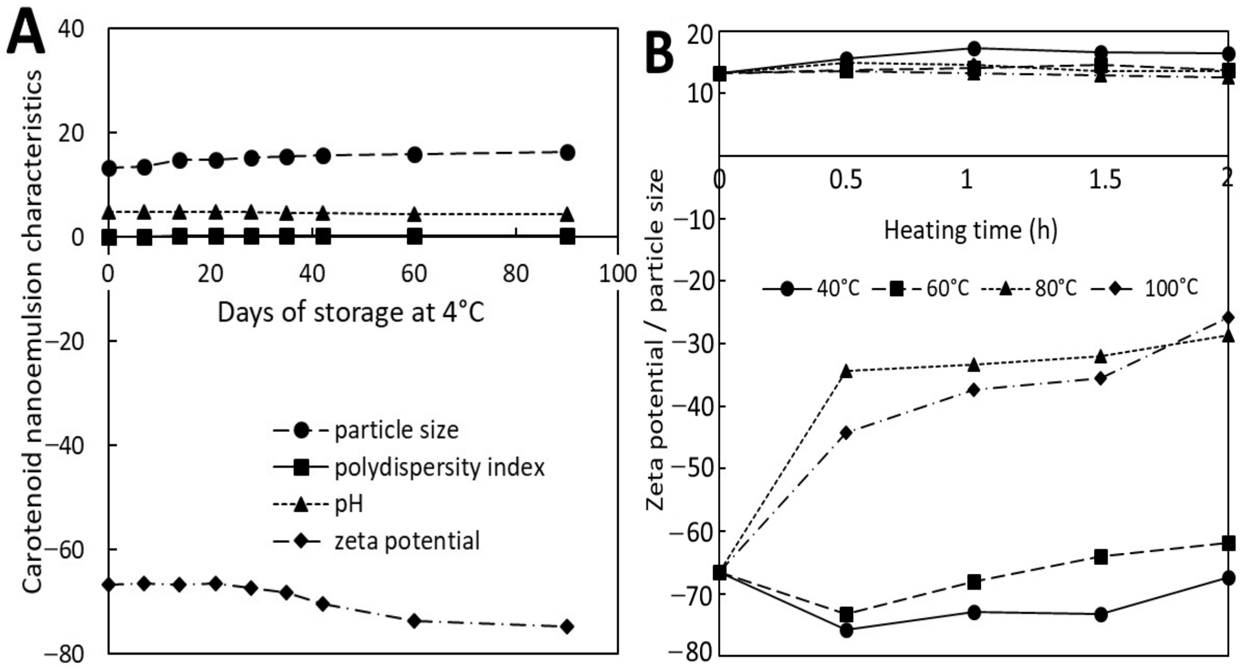

Figure S1. Particle size, polydispersity index, pH and zeta potential of carotenoid nanoemulsion as affected by storage at 4°C for 90 days (A) as well as both particle size and zeta potential of carotenoid nanoemulsion as affected by heating at different times and temperatures (B).
